# The prevalence and prognostic value of diabetes and hypertension in patients treated for cholera during the ongoing Syrian conflict

**DOI:** 10.1016/j.clinpr.2024.100362

**Published:** 2024-07

**Authors:** Ibrahim Antoun, Alkassem Alkhayer, Ahmed Kotb, Joseph Barker, Alamer Alkhayer, Yaman Mahfoud, Riyaz Somani, G. André Ng, Aya Tarraf, Daniel Pan

**Affiliations:** a Department of Cardiovascular Sciences, Clinical Science Wing, Glenfield Hospital, Leicester, UK; b University of Tishreen, Latakia, Syria; c National Hospital, Latakia, Syria; d Department of Cardiology, University Hospitals of Leicester NHS Trust, Glenfield Hospital, Leicester, UK; e NIHR Leicester Biomedical Research Centre, UK; f Department of Respiratory Sciences, University of Leicester, UK; g Development Centre for Population Health Sciences, University of Leicester, UK; h Department of Infectious Diseases and HIV Medicine, University Hospitals of Leicester NHS Trust, UK; i Li Ka Shing Institute of Health Information and Discovery, Oxford Big Data Institute, UK; j WHO Collaborating Centre for Infectious Diseases Epidemiology and Control, School of Public Health, University of Hong Kong, Hong Kong

**Keywords:** Cholera, Syria, Earthquake, Conflict, Multimorbidity, Clinical outcomes

## Abstract

**Objectives:**

Little work has been done investigating the prevalence and impact of comorbidities on cholera outcomes within conflict settings. We describe the clinical outcomes of patients treated for cholera in Latakia, Syria, during the 2022–2023 cholera outbreak.

**Methods:**

We performed a single-centre retrospective observational cohort study of patients admitted to the hospital with suspected cholera between 15th December 2022 and 15th February 2023 at National Hospital, Latakia, Syria. Multivariable negative binomial regression analysis was used to investigate the variables’ relationship to hospital length of stay.

**Results:**

The study involved 89 patients admitted to the hospital; none were vaccinated against cholera. Cholera rapid diagnostic test was positive in 91 % of patients. Half of the patients (51 %) were male; a third (35 %) had hypertension, and a fifth (19 %) had diabetes. One patient passed away during admission. Patients who were male, had diabetes and were hypertensive tended to have longer hospital stays in univariable analysis. When these factors were added to a multivariable negative binomial model (consisting of age, gender, hypertension and diabetes), male sex (IRR: 4.1, 95 % CI: 1.28–6.2, *p* = 0.001), the presence of hypertension (IRR: 2.1,95 % CI: 1.14 to 4.1, *p* = 0.004) and diabetes (IRR: 2, 95 % CI: 1.2 to 2.7, *p =* 0.001) were independent predictors of longer hospital stays.

**Conclusion:**

In conflict settings, hypertension and diabetes are common in patients admitted to hospital with cholera and are associated with longer hospital stays. Mortality from cholera, however, was low. Our findings highlight the importance of comorbidity control in mitigating excess morbidity during infectious disease outbreaks within conflict areas.

## Highlights

• We describe the clinical outcomes of patients treated for cholera in Syria between December 2022 and February 2023.
• Of 89 patients admitted to hospital for cholera, 35 % had hypertension and 19 % had diabetes.
• Having hypertension and diabetes was associated with longer lengths of stay for these patients.
• Our findings highlight the importance of comorbidity control to mitigate excess morbidity in infectious disease outbreaks.

## Introduction

An outbreak of cholera, the acute watery diarrheal illness caused by toxigenic *Vibrio cholerae,* has been reported in Syria for the first time in 15 years, starting in August 2022 (Devi, 2022). In February 2023, 193,848 suspected Cholera cases with 52 mortalities have been reported in Northern Syria alone (EWARN, 2023, Syrian Arab republic, 2023). Cholera outbreaks can happen during emergencies like floods and earthquakes or when water supply, sanitation, and hygiene (WASH) infrastructure is deficient. The outbreak’s source is thought to be a consequence of the civil war and concerted interference with (WASH), resulting in water contamination from the Euphrates River, used for drinking and irrigating crops, leading to food contamination (Abbara et al., 2022).

Before the outbreak, Syria has been suffering civil war since 2011, damaging two-thirds of the country’s water treatment plants, half of its pumping stations and a third of its water towers. At least 70 % of the country’s sewage is untreated (Abbara et al., 2022). Conflict and lack of healthcare funding, which escalated during the COVID-19 pandemic, has left less than half of its hospitals fully functioning, with over 50 % of its healthcare workforce forced to leave due to conflict. Thus, many citizens remain unvaccinated against cholera, highly exposed to the pathogen and at risk of symptomatic cholera illness. The recent earthquake in February 2023 in Syria and Turkey also negatively affected the outbreak response by limiting access to healthcare services and reducing funds (Whole of Syria Cholera Outbreak Situation Report no. 13 Issued 28 February, 2023).

Many studies have explored risk factors for acquiring symptomatic cholera in outbreak settings. For example, a recent *meta*-analysis involving 32 studies has proposed that the risks of contracting cholera are environmental factors, socioeconomic factors, specific genetic features, and immunodeficiency (Richterman et al., 2018). However, few have assessed factors relating to severe disease from cholera once someone is infected, especially within conflict settings. In particular, the prevalence and impact of multiple long-term conditions, such as diabetes and hypertension, on cholera remains unknown.

We aim to describe the prevalence and impact of multiple long-term conditions on the treatment and clinical outcomes relating to patients treated for cholera within the Syrian conflict. Given the poor infrastructure and lack of access to regular physician care, we hypothesised that multiple long-term conditions would negatively outcomes from cholera infection. We sought to see whether having multiple long-term conditions was associated with morbidity from cholera after taking into account other risk factors.

## Methods

### Study design and participants

We performed a single-centre retrospective observational cohort study at the National Hospital, Latakia, Syria. Latakia is Syria’s 4th largest city in the Mediterranean region. The National Hospital in Latakia is a public hospital funded by the Syrian Ministry of Health; it has around 320 beds, an emergency department, and an intensive care unit of 20 beds. The study involved adult patients (over 16 years of age) presenting to the hospital with suspected cholera between the 15th of December 2022 and the 15th of February 2023. Patients were followed up until discharge or death. The diagnosis was made on clinical suspicion from the presentation with acute water diarrhoea (AWD) and confirmed by stool culture showing *Vibrio cholera *serogroup O1 or O139 if resources allow. Crystal® VC Rapid Diagnostic Test (RDT) was done in all patients to aid in diagnosis. If RDT is positive and resources are available, the diagnosis is confirmed by stool culture if resources allow. Antibiotic sensitivity testing for culture-positive patients was not done due to the paucity of resources during the outbreak and earthquake period. Clinical and laboratory characteristics were collected from the patient notes. The primary outcomes were length of stay in the hospital and in-hospital mortality. The project was conducted as an audit in the institution (approval reference: 2023/212A).

### Statistical analysis

Continuous variables are expressed as median and interquartile ranges (IQR), which include age, length of hospital stay, lab results, and vital signs. Categorical variables are expressed as numbers and percentages (%), including gender, presenting complaints, comorbidities, and treatments given. Fisher's exact test was used to compare two groups when the outcome is a categorical variable. These variables included gender and comorbidities. The student's *t*-test was used to compare continuous variable means between the two groups after confirming that the data follows normal distribution using the Shapiro-Wilk test. The variables studied included age, length of hospital stay, lab results and vital signs.

Given that only one patient died in our cohort, we used negative binominal regression to investigate the relationship between variables and hospital length of stay. Our multivariable model was constructed *a priori* and included age, gender and comorbidities associated with hospital length of stay on univariable analysis. The incident rate ratio (IRR) estimates are shown with 95 % CIs. A 2-sided *p-value* < 0.05 was considered statistically significant. All p values are two-sided and are presented without adjustment for multiple testing. Epidemic curves illustrate the number of patients presenting to the hospital with cholera over time. Statistical analysis was performed using GraphPad Prism V9.5 for Mac (San Diego, California, USA; www.graphpad.com).

## Results

**Fig. 1:**
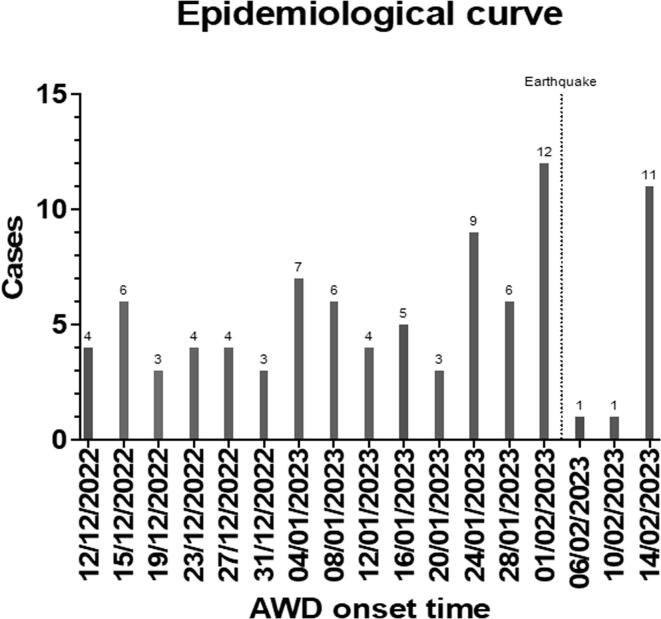
Epidemiological curve of cholera admissions over a 2-month period in the study centre, including the day of the earthquake on 06/02/2023. AWD: acute watery diarrhoea.

Between 15th December 2022 and 15th February 2023, 89 patients presented to the hospital and were treated for cholera. All 89 patients suspected to have cholera were admitted to the hospital for treatment after being reviewed by a medical consultant. Fig. 1 shows the epidemic curve of patients presenting to the hospital with suspected cholera over our study period. We observed that patients presented increasingly large waves during the cholera epidemic despite a temporary drop in cases in the first two weeks of February 2023 following an earthquake. None of the patients in our cohort were from refugee camps, reported recent travel outside Latakia within the last month or had been vaccinated against cholera. The age range for patients admitted to hospital with cholera was 17 to 69 years old. No children were admitted during this time.

Regarding admission details and patient demographics, we found that patients presented rapidly following symptom development (median duration of symptoms 2 days, IQR 2–3, Table 1). Besides diarrhoea, other presenting symptoms included vomiting (n = 15, 17 %) and fever (n = 7, 8 %). Half of the cohort were male. We found that within this relatively young cohort (median age: 41 years, IQR 23–59), a fifth of the cohort had diabetes and a third had hypertension. Upon presentation to the hospital, most patients were clinically dehydrated, with clinical tachycardia and hypotension, as well as elevated urea and lactate and low serum potassium on laboratory investigations (Table 1).

**Table 1:** Patient details, admission, vital signs and lab workup stratified by admission time.

Variable	Full cohort (n = 89)	>2 days (n = 39)	≤2 days (n = 50)	p-value
Demographics				
Age (years)	41 (23–59)	50 (23–61)	35 (22–54)	0.25
Male	45 (51 %)	33 (66 %)	12 (31 %)	0.001
Hypertension	31 (35 %)	21 (42 %)	10 (26 %)	0.012
Diabetes mellitus	17 (19 %)	14 (28 %)	3 (8 %)	0.03
Symptoms duration (days)	2 (2–3)	2 (1–4)	2 (2–3)	0.58
Ischemic heart disease	3 (3 %)	1 (2 %)	2 (5 %)	0.58
Chronic lung disease	3 (3 %)	2 (4 %)	1 (3 %)	0.99
Chronic kidney disease	10 (11 %)	8 (16 %)	2 (5 %)	0.18
Lab values				
Haemoglobin (g/dL)	12 (10–14)	12 (10–14)	12 (10–14)	0.71
C-reactive protein (mg/dL)	54 (31–83)	50 (28–77)	67 (33–89)	0.60
Urea (mg/dL)	14 (11–19)	15 (11–20)	14 (10–18)	0.32
Potassium (mmol/L)	3.7 (3.2–4.1)	3.4 (2.9–3.9)	3.8 (3.2–4.2)	0.13
White cell count (10^9^/L)	14 (11–17)	14 (11–18)]	12 (9–17)	0.19
Bicarbonate (mmol/L)	19 (12–22)	19 (12–21)	19 (12–22)	0.39
Creatinine (µmol/L)	141 (104–193)	135 (104–186)	159 (104–195)	0.77
Lactate (mmol/L)	3 (2–4.8)	3.1 (1.8–4.4)	2.8 (2–5)	0.68
Vital signs				
Heart rate (beats per minute)	115 (100–129)	112 (99–128)	116 (100–129)	0.36
Temperature (°C)	37 (36–37)	37 (37–37)	37 (37–37)	0.49
Respiratory rate (respirations per minute)	16 (14–18)	16 (14–17)	16 (13–18)	0.83
Blood pressure (systolic/diastolic) (mmHg)	99 (82–110)59 (52–70)	98 (82–110) 59 (50–69)	99 (84–100) 64 (53–71)	0.5/0.3

Student *t*-test was used to compare means of vital signs, age and lab values. Fisher's exact test was used to compare gender and comorbidities.

We observed that cholera RDT was positive in most patients (n = 81, 91 %); the remainder were treated for cholera based on high clinical suspicion. Due to limited resources, stool culture was only feasible in five patients, all of which were positive. All patients received combined oral rehydration solution (ORS) and intravenous hydration therapy in the form of crystalloid fluids administered on admission with 3 (3–4) litres in the first 24 h. Normal isotonic saline was the most common crystalloid fluid in 78 patients (88 %). Dextrose 5 % was administered in 20 patients (22 %), with 9 (10 %) patients having it in combination with saline. Intravenous bicarbonate replacement occurred in 14 patients (16 %). No colloid fluids were administered.

All patients received empirical antibiotics as a part of their cholera treatment plan. Antibiotics choice was based on stock availability. Ciprofloxacin 500 mg twice daily was the most common antibiotic in 72 patients (81 %), with 59 (66 %) combined with metronidazole 400 mg three times a day. Azithromycin 500 mg once a day was used in 12 patients (13 %), a combination with ceftriaxone 2 g once a day and levofloxacin 500 mg twice daily was used in five patients (6 %). Metoclopramide 10 mg has been given to 33 patients for sickness as needed, a maximum of four times a day (37 %). Organ support was required in three patients (3 %) through dialysis. One patient passed away because of multiorgan failure after 16 days of admission and required urgent dialysis by the intensive care unit due to hypovolemic shock and anuria on the background of chronic kidney disease.

Amongst our cohort, the median length of stay in the hospital was 3 days (IQR:2–4). We found that male patients, as well as those with diabetes and hypertension, tended to have longer hospital stays (Table 1 and Table 2). When these factors were added into a multivariable negative binomial regression model consisting of age, gender, hypertension and diabetes, male gender (IRR:4.1, 95 % CI: 1.28–6.2, p = 0.001, hypertension (IRR:2.1,95 % CI: 1.14 to 4.1, p = 0.004) and diabetes (IRR:2, 95 % CI: 1.2 to 2.7, p = 0.001; Table 2) remained independent factors associated with longer hospital stay.

**Table 2:** Negative binomial regression and univariate multivariate models examining the effect univariate analysis on length of admission in patients with cholera. Statistically significant variates in the univariate analysis were examined in multivariate analysis along with a base model consisting of age and sex. This was added to a base model which included age and sex.

Univariable analysis				Multivariable analysis		
Variables	IRR	95% CI	P-value	IRR	95% CI	P-value
Age (years)	1	0.9 to 1	0.62	1	0.99 to 1	0.45
Male (yes vs no)	2.2	1.4 to 3.6	0.001	4.1	1.28 to 6.2	<0.001
Hypertension (yes vs no)	2.1	1.3 to 3.5	0.002	2.1	1.14 to 4.1	0.004
Diabetes mellitus (yes vs no)	2.4	1.4 to 4.2	0.001	2	1.2 to 2.7	0.001
Symptoms duration (per day increase)	0.97	0.8 to 1.2	0.78			
Ischemic heart disease (yes vs no)	1.4	0.6 to 1.4	0.64			
Chronic lung disease (yes vs no)	1.1	0.3 to 4	0.91			
Chronic kidney disease (yes vs no)	0.88	0.4 to 1.9	0.74			
Volume within first 24 hours (per 1 litre increase)	0.97	0.8 to 1.2	0.77			
Haemoglobin (per unit- g/dL increase)	1	0.9 to 1	0.85			
C-reactive protein (per unit- mg/dL increase)	1	0.9 to 1	0.69			
Urea (per unit- mg/dL increase)	1	0.9 to 1	0.53			
Potassium (per unit- mmol/L increase)	0.86	0.8 to 1	0.23			
White cell count (per unit-10^9^/L increase)	0.96	0.9 to 1	0.1			
Bicarbonate (per unit- mmol/L increase)	1	0.9 to 1	0.23			
Creatinine (per unit- mmol/L increase)	1	0.9 to 1	0.67			
Lactate (per unit- mmol/L increase)	1.1	0.9 to 1.3	0.68			
Heart rate (per beat per minute increase)	1.2	0.8 to 1.4	0.66			
Temperature (per unit-°C increase)	1.1	0.8 to 1.4	0.49			
Respiratory rate (per unit- respiration per minute increase)	1	0.9 to 1	0.53			
SBP/DBP (per unit-mmHg increase)	1*	0.9 to 1*	0.48/0.65			

IRR: incidence rate ratio.

* Both systolic blood pressure and diastolic blood pressure showed similar results.

## Discussion

We report an inpatient cohort of 89 patients treated for cholera infection in Syria. Within outbreak settings, we show that most patients who present to the hospital with cholera survived. However, we found that cardiovascular comorbidities, such as diabetes and hypertension, were common within our relatively young cohort and related to longer hospital lengths of stay.

We note that RDT did not guide cholera treatment amid an outbreak but by clinical judgment. Nevertheless, RDT was positive in 91 % of suspected cases in our cohort. Although our cohort is relatively small, similar results were found in a recent *meta*-analysis of 17 studies and 12,627 that mainly utilised the same RDT used in our cohort and showed a comparable sensitivity of 91 % and specificity of 80 % (Muzembo et al., 2022). On an individual level, it is unlikely that treatment will be dictated by a cholera RDT. Still, we have demonstrated the utility of these tests in constructing epidemic curves, which are essential for documenting the natural history of the outbreaks.

Although cholera epidemics were associated with high mortality, our cohort demonstrated much lower mortality (<1%) compared to a Sierra Leone-based study involving 798 during their outbreak in 2012 with a mortality rate of 2.3 %. It is noted that antibiotics were only used in half the patients compared to all patients in our cohort, which was in keeping with the Centre for Disease Control and Prevention (CDC) recommendations (Blacklock et al., 2015). Our mortality rate was also less than the Malawi outbreak, as declared by the Ministry of Health (3.3 %), with most mortality occurring in patients over 60. No comorbidity profile or treatment plans were provided in the statement. However, it is possible that the relatively younger age in our cohort contributed to favourable outcomes (Wise, 2023). Our outcomes, however, were similar to the North-East Nigeria outbreak in 2019 (<1%) in 9,725 (Fagbamila et al., 2023). The study did not comment on management, but half the patients affected were between 5 and 20 years old, contributing to favourable outcomes. Interestingly, none of our inpatients were younger than 17 years old. It may be that in our population, infected children had less severe illness, not requiring hospitalisation. Differences in clinical outcomes may be attributable to the availability of fluid hydration therapy and timely administration of broad-cover antibiotics.

We found that diabetes and hypertension were common in our patients and predicted length of stay in hospital. There is increasing evidence to suggest the convergence of infectious and non-communicable disease epidemics within lower-income countries rather than the classic epidemiological transition in which societies shift from predominantly infectious to predominantly non-communicable disease epidemics (Wong et al., 2021 Jul 1). Prolonged diarrhoea can lead to dehydration and electrolyte imbalances, exacerbating diabetic complications (Al-Sharafi et al., 2020). Severe dehydration caused by cholera can also strain the cardiovascular system, potentially exacerbating existing hypertension. Within conflict settings, many patients may not be on adequate prognostic cardiovascular medications; thus, it is likely that their diabetes and hypertension were uncontrolled before becoming infected with cholera (Al-Sharafi et al., 2020). On the other hand, diuretics used to treat hypertension (thiazide diuretics) can also lead to further fluid loss and worsening dehydration. Finally, cardiovascular diseases, significantly if inadequately controlled, can contribute to inflammation, which in turn is exacerbated by an active infection (Jaén et al., 2020). Whilst the length of stay may not necessarily be a marker of the severity of infection, it is a commonly used outcome measure for hospital studies. If the length of stay decreases, fewer hospital resources are consumed, and care becomes more efficient and effective. A shorter stay also reduces the probability of nosocomial transmission of cholera.

Our study highlights the importance of having a robust healthcare system that can address the emerging issue of multiple long-term conditions within cohorts that experience conflict (Table 1). This would not only benefit the treatment of long-term conditions themselves but also mitigate morbidity from acute infections. Of note, none of our cohort was vaccinated against cholera. This is another potential intervention that could have a significant effect on morbidity.

Our study had limitations. Our data collection was limited to a single centre and included only routinely collected data within the medical records and by the number of patients who presented to the hospital. No cholera serotype was provided for culture-positive patients. This study was based on an emergency response under challenging circumstances. Therefore, specific criteria for discharge and adherence to cardiovascular medications could not be ascertained. Other comorbidities may also contribute to morbidity, but due to limited resources, they were underdiagnosed in our cohort. Nevertheless, our study provides significant, timely findings that others may find helpful for future outbreaks.

In conclusion, we found that patients presenting to the hospital in Syria with cholera had a high prevalence of diabetes and hypertension and low mortality rates compared to other Cholera outbreaks. Male sex, diabetes and hypertension were independently associated with longer hospital stays for Cholera after taking into account age and sex. Our findings have urgent public health implications for identifying and treating multiple long-term conditions within conflict settings. Future studies should focus on Cholera outcomes and management in other Syrian Centres to assess the generalisability of these results.

## Declaration of competing interest

The authors declare that they have no known competing financial interests or personal relationships that could have appeared to influence the work reported in this paper.
